# The Clinical Analysis of Checkpoint Inhibitor Pneumonitis with Different Severities in Lung Cancer Patients: A Retrospective Study

**DOI:** 10.3390/jcm13010255

**Published:** 2024-01-01

**Authors:** Hui Huang, Ruxuan Chen, Yan Xu, Nan Fang, Chi Shao, Kai Xu, Mengzhao Wang

**Affiliations:** 1 Department of Pulmonary and Critical Care Medicine, Peking Union Medical College Hospital, Chinese Academy of Medical Sciences and Peking Union Medical College, Beijing 100730, China; pumchhh@126.com (H.H.); chenruxuan@pumch.cn (R.C.);; 2 State Key Laboratory of Bioactive Substance and Function of Natural Medicines, Institute of Materia Medica, Chinese Academy of Medical Sciences and Peking Union Medical College, Beijing 100050, China; 3 Department of Radiology, Peking Union Medical College Hospital, Chinese Academy of Medical Sciences and Peking Union Medical College, Beijing 100730, China

**Keywords:** immune-related adverse events, checkpoint inhibitor pneumonitis, severity grade, prognosis

## Abstract

Immune-related adverse events (irAEs) of immunotherapy would lead to the temporary or permanent discontinuation of immune checkpoint inhibitors (ICIs). Among them, checkpoint inhibitor pneumonitis (CIP) is a potentially life-threatening irAE. This study aimed to identify the differences between patients with low-grade CIPs (grades 1–2) and high-grade CIPs (grades 3–5) and to explore the prognostic factors. We retrospectively reviewed the medical records of 916 lung cancer patients who were treated with ICIs. Patients with CIPs were identified after multidisciplinary discussion, and their clinical, laboratory, radiological, and follow-up data were analyzed. Among the 74 enrolled CIP patients, there were 31 low-grade CIPs and 43 high-grade CIPs. Compared with low-grade CIP patients, patients with high-grade CIPs were older (65.8 years vs. 61.5 years) and had lower serum albumin (35.2 g/L vs. 37.9 g/L), higher D-dimer (5.1 mg/L vs. 1.7 mg/L), and more pulmonary infectious diseases (32.6% vs. 6.5%) during follow-up. In addition, complication with pulmonary infectious diseases, management with intravenous immunoglobulin, tocilizumab, and longer duration of large dosage corticosteroids might be associated with worse outcomes for patients with CIPs. This study highlights potential risk factors for high-grade CIP and poor prognosis among lung cancer patients who were treated with anti-cancer ICIs.

## 1. Introduction

The prognosis of patients with lung cancer has improved with the administration of immune checkpoint inhibitors (ICIs). However, the activation of the host immunity by ICIs would cause immune-related adverse events (irAEs) during the anti-cancer immunotherapy, which would lead to the temporary or permanent discontinuation of ICIs. Sometimes, irAEs might be life-threatening, permanent, and/or require long-term immunosuppressant administration, which would be associated with a poor prognosis. Checkpoint inhibitor pneumonitis (CIP) is a relatively uncommon but serious, potentially life-threatening irAE. The overall incidence of CIP was 2.7% for monotherapy and 6.6% for combination therapy in programmed cell death protein 1 (PD-1) inhibitor therapy clinical trials [1], and CIP was more common in patients with non-small cell lung cancer (NSCLC) than in melanoma [2]. Among patients with NSCLC, it was reported that individuals with squamous cell lung carcinoma encountered a more frequent incidence of CIP during ICIs treatment than those with lung adenocarcinoma. Pre-existing interstitial lung disease or diffuse emphysema and combination anti-cancer therapy, including radiotherapy, chemotherapy, or small-molecule targeted therapy, were also potential risk factors for CIP. The overall incidence of grade 3–5 and grade 5 irAEs during ICIs therapy was 30.5% and 1.1%, respectively. It is worth noting that CIP was the most common cause of grade 5 irAEs, both in monotherapy (36.7%) and combination therapy (21.1%) [3]. On the other hand, it was reported that the occurrence of irAEs was associated with survival efficacy for cancer patients prescribed ICIs therapy [4,5]. Therefore, it is urgent to improve the prognosis of CIP, especially for patients with lung cancer.

With the improvement of treatment for CIPs, most patients with grade 1–2 CIPs and many patients with grade 3–4 CIPs would recover after suspension of ICIs and/or corticosteroids administration. Furthermore, some of them could be rechallenged with ICIs successfully [6,7,8]. However, pre-existing lung diseases, steroid-refractory CIPs, complications with secondary pulmonary infectious diseases, and/or progression of underlying lung cancer were common poor outcomes for patients with CIPs, especially for those with high-grade CIPs [9,10,11,12]. Therefore, in this retrospective study on patients with lung cancer, we aimed to identify different features between patients with low-grade CIPs and high-grade CIPs and to explore potential prognostic factors.

## 2. Materials and Methods

### 2.1. Patients

There were 2302 patients with pathology-confirmed kinds of malignancies who were administered ICIs at Peking Union Medical College Hospital wards from 1 July 2018 to 1 December 2021. Among them, there were 916 patients with lung cancer. The final follow-up point was 30 June 2022. The median follow-up period was 190 days [interquartile range (IQR) 83.25 days, ranging from 8 to 1321 days]. Follow-up information was obtained through outpatient follow-up records or telephone conversations with patients or their families.

**Figure 1:**
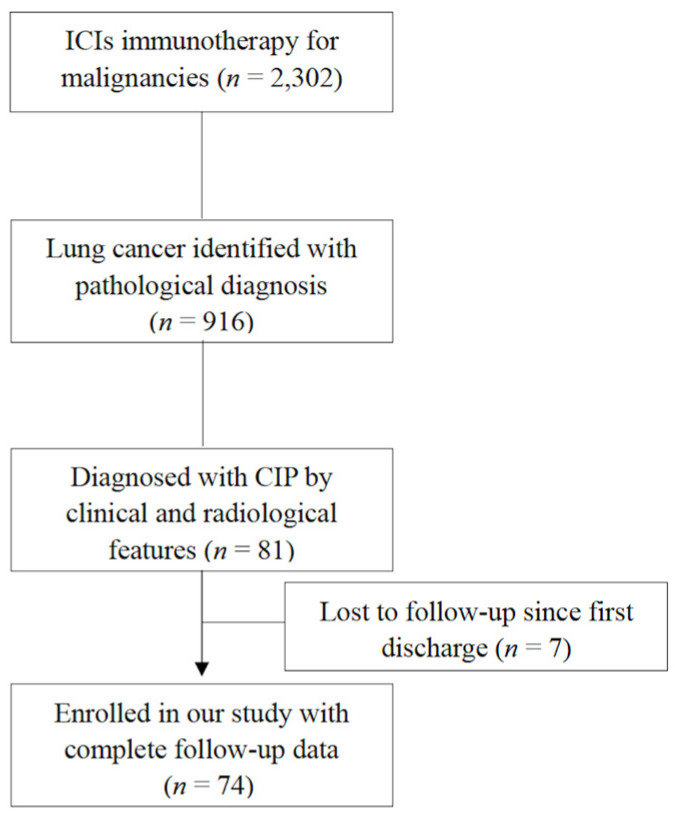
This study flow chart.

After two researchers reviewed their medical records (including their pathological reports, C.S. and Y.X.) and chest computed tomography (CT) images from the hospital data bank (R.C., K.X. and H.H.), 81 patients with CIPs were identified. However, seven patients have been lost to follow-up since first discharge. Finally, 74 CIP patients were enrolled in our study with complete clinical and follow-up information and chest CTs in our hospital data bank. (Figure 1 shows this study flow chart).

### 2.2. Definitions

The diagnosis of CIP was made after an exclusive diagnostic evaluation: (1) new onset or exaggeration of respiratory manifestations, especially of dry cough and dyspnea; a decrease in oxygen saturation (measured by a finger pulse oxygen saturation detector) after ICIs immunotherapy, with or without fever; (2) new pulmonary shadows characterized by the presence of hallmark manifestations visualized by CT imaging [13]; and (3) absence of evidence of pulmonary infectious disease, lung cancer progression, and/or pulmonary edema.

The diagnosis of pulmonary infectious diseases was made with respiratory sample culture, specific staining, polymerase chain reaction testing, next-generation sequencing, and/or specific serum biomarkers.

The grades of CIPs were classified by the commonly used National Cancer Institute’s Common Terminology Criteria for Adverse Events (CTCAE) in the first CIPs admission: asymptomatic/mild (grade 1), moderate (grade 2), severe (grade 3), life-threatening (grade 4), and death (grade 5) [14,15]. Patients with grade 1 and grade 2 CIPs were defined as low-grade CIPs, and patients with grade 3 to grade 5 CIPs were defined as high-grade CIPs.

There were five kinds of outcomes for our enrolled patients with CIPs, including cured, improved, stable, deteriorated, and death. Among them, patients who were cured, improved, or stable after treatment were included in the better prognosis group, and the rest were defined as the worse prognosis group.

### 2.3. Ethics

This study was approved by the institutional ethical review board (IRB) of Peking Union Medical College Hospital (approval number: K2135) in accordance with the Declaration of Helsinki. Written informed consent from each patient was waived because our study was conducted using anonymized health care data, which met the IRBs minimal risk waiver criteria.

### 2.4. Statistical Analysis

The data were analyzed using the SAS version 9.4 software package (SAS Institute Inc., SAS Campus Drive, Cary, NC 27513, USA). GraphPad Prism version 8.0 (GraphPad Software, San Diego, CA, USA) was used for graphing. The continuous variables were presented as the means ± standard deviation (SD) or median (IQR) values, and the categorical variables were presented as frequencies and percentages. The *t*-test or rank sum test was used for continuous variables, and the chi-square test was used for categorical variables. A two-tailed *p* < 0.05 was considered statistically significant. The log-rank test was used to compare the survival rates of different subgroups.

## 3. Results

### 3.1. Clinical Characteristics of All Enrolled CIP Patients

In total, 74 CIPs patients were enrolled in our study. There were 58 males (78.4%) and 16 females (21.6%). The average age at the diagnosis of lung cancer was 62.89 ± 8.64 (range: 38–78) years, and the majority (52 patients, 70.3%) were older than 60 years. When they suffered from CIPs, the average age was 64 ± 8.38 (range: 39–78) years. More than half of the patients (48 patients, 64.9%) had a smoking history. There were 31 patients (41.9%) with emphysema on chest CT imaging.

All the enrolled patients were diagnosed pathologically with lung cancer. Among them, there were 32 patients (43.2%) with adenocarcinoma, 31 patients (41.9%) with squamous cell cancer (SCC), 6 patients (8.1%) with small cell lung cancer (SCLC), and 5 patients (6.8%) with other lung cancers. Forty-eight patients (64.9%) were diagnosed with stage IV lung cancer, 25 patients (33.8%) were stage III, and one patient (1.4%) was stage II.

**Figure 2:**
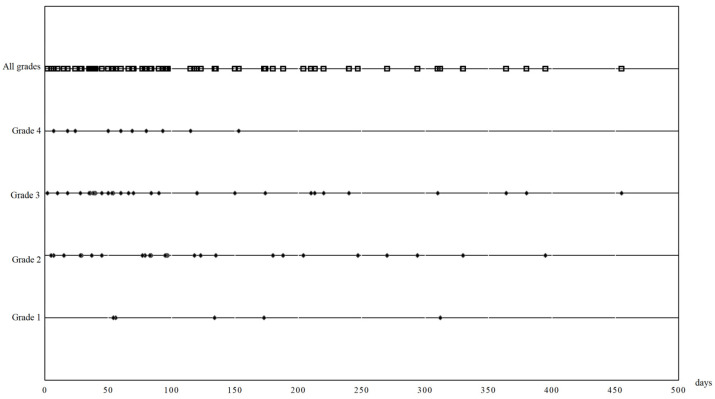
The timepoint of checkpoint inhibitor pneumonitis onset since the beginning of immune checkpoint inhibitor therapy The grade of CIPs was classified by the commonly used National Cancer Institute’s Common Terminology Criteria for Adverse Events (CTCAE) on the first CIP admission.

The median time to onset of CIP was 83 days (2.77 months, range 2–455 days, Figure 2) after the first dose of ICIs. According to CTCAE, there were 5 patients (8.1%) with grade 1 CIPs, 26 patients (35.1%) with grade 2 CIPs, 33 patients (44.6%) with grade 3 CIPs, and 10 patients (3%) with grade 4 CIPs.

**Figure 3:**
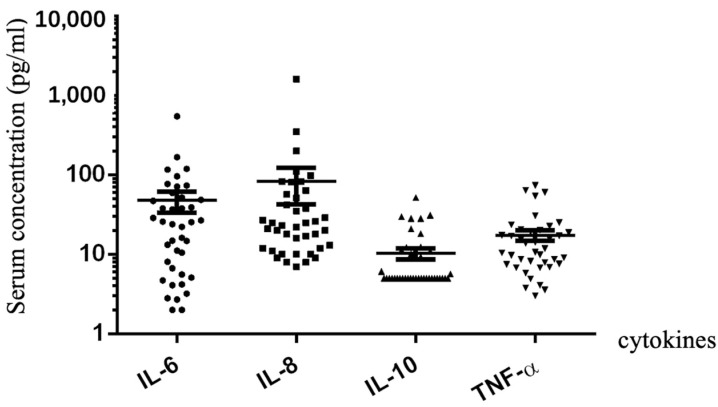
The serum concentration of cytokines at the onset of checkpoint inhibitor pneumonitis, including interleukin (IL)-6, IL-8, IL-10, and tumor necrosis factor (TNF)-α.

Serum cytokine analysis was not routinely ordered for lung cancer patients during ICIs treatment. In this study, there were 40 patients who underwent serum cytokine analysis for the diagnosis of CIPs, including interleukin (IL)-6, IL-8, IL-10, and tumor necrosis factor (TNF)-α. The median serum concentration of IL-6, IL-8, IL-10, and TNF-α was 25.7 pg/mL (range: 2–542 pg/mL; normal reference range: <5.9 pg/mL; 30 patients/75% with elevated serum IL-6), 24 pg/mL (range: 7–1603 pg/mL; normal reference range: <62 pg/mL; 9 patients/34.7% with elevated serum IL-8), 5 pg/mL (range: 5–51.9 pg/mL; normal reference range: <9.1 pg/mL; 13 patients/32.5% with elevated serum IL-10), and 12.9 pg/mL (range: 3–73.7 pg/mL; normal reference range: <8.1 pg/mL; 28 patients/70% with elevated serum TNF-α), respectively. The serum cytokine analysis is shown in Figure 3. There were only 13 patients with low-grade CIPs and 14 patients with high-grade CIPs who were arranged with bronchoalveolar lavage as they suffered from CIPs.

Corticosteroids, intravenous immunoglobulin (IVIg), tocilizumab, and antibiotics were the common medications prescribed for CIP patients, especially for high-grade CIPs [12]. Differences in medication management between CIP patients with different outcomes are shown in Table 1. The administration duration of methylprednisolone > 80 mg/d (8.82 days vs. 3.14 days, t = 3.39, *p* = 0.001) and duration of prednisolone > 1 mg/kg/d (18.5 days vs. 9.7 days, t = 3.26, *p* = 0.002) were longer in patients with a worse prognosis. Patients with worse prognosis were predisposed to be administered IVIg (90.9% vs. 7.9%, χ^2^ = 9.57, *p* = 0.004), tocilizumab (36.4% vs. 7.9%, χ^2^ = 7.08, *p* = 0.02), antibiotics (100% vs. 65.1%, χ^2^ = 5.47, *p* = 0.03), and anti-fungal medications (45.5% vs. 7.9%, χ^2^ = 11.3, *p* = 0.005), respectively.

### 3.2. Clinical Characteristics of Patients in the Low-Grade CIPs Group vs. the High-Grade CIPs Group

The clinical characteristics of patients in the low-grade CIPs group vs. the high-grade CIPs group are shown in Table 2 and Table 3, including the clinical features at baseline (before CIPs) and at the time of CIPs. The patients with high-grade CIPs were older than those with low-grade CIPs (65.8 ± 6.5 years vs. 61.5 ± 10.1 years, t = 2.21, *p* = 0.03). Male was more common in both the low-grade and high-grade CIP groups (77.4% vs. 79.1%, t = 0.03, *p* > 0.05). There were more patients with worse performance status both before ICIs therapy (t = 1.22, *p* = 0.047) and at the time of CIPs (t = 17.9, *p* = 0.001) in the high-grade CIPs group. Some lung cancer patients had underlying autoimmune diseases, interstitial lung diseases (ILDs), other malignancies, and diabetes, which might be risk factors for CIPs when they were prescribed with ICIs. However, there were no statistically significant differences in comorbidities between low-grade and high-grade CIP groups. The baseline characteristics of lung cancers, including the distributions of pathological patterns of lung cancer (χ^2^ = 6.25, *p* = 0.18), the surgical resection rate (χ^2^ = 2.26, *p* = 0.32), radiotherapy (χ^2^ = 0.48, *p* = 0.49), molecular targeting therapy (χ^2^ = 0.02, *p* = 0.11), and chemotherapy (χ^2^ = 0.02, *p* = 0.90), were also similar between the two groups. The ICIs might be prescribed as different lines of therapy for patients with lung cancer, e.g., adjuvant therapy, maintenance therapy, first line, second line, third line, etc. There were no significant differences in ICIs lines between low-grade and high-grade CIPs groups (χ^2^ = 7.42, *p* = 0.19).

Fever was more common in patients with high-grade CIPs (65.1% vs. 29%, t = 9.38, *p* = 0.002). Elevated serum lactate dehydrogenase (LDH) [48/64.9%, (325.8 ± 466.2) U/l], C-reaction protein (CRP) [67/90.5%, (60.9 ± 54.2) mg/L], erythrocyte sedimentation rate (ESR) [68/91.9%, (56.6 ± 32.3) mm/h], D-dimer [66/89.2%, (3.78 ± 9.68) mg/L] and decreased serum albumin [31/41.9%, (36.3 ± 40.8) g/L] were common in CIPs patients. And patients with high-grade CIPs showed lower serum albumin [(35.2 ± 4.2) g/L vs. (37.9 ± 4.6) g/L, χ^2^ = 2.59, *p* = 0.01] and higher D-dimer [(5.1 ± 7.1) mg/L vs. (1.7 ± 2.2) mg/L, χ^2^ = 2.36, *p* = 0.02). There were no significant differences in CRP [(45.8 ± 48.1) mg/L vs. (68.5 ± 56.8) mg/L, χ^2^ = 1.53, *p* = 0.13), ESR [(56.6 ± 32.3) mm/h vs. (63 ± 33.1) mm/h, χ^2^ = 0.63, *p* = 0.53), or LDH [(286.1 ± 103.7) U/l vs. (350.1 ± 157) U/l, χ^2^ = 1.74, *p* = 0.09].

Most patients (70/94.6%) showed new onset shadows in bilateral lungs when they suffered from CIPs, and nonspecific interstitial pneumonia (NSIP) (33/44.6%) and/or organizing pneumonia (OP) patterns (30/40.5%) were the common radiological patterns. After reviewing their chest CTs, we found there were no significant differences in emphysema (32.2% vs. 48.8%, t = 2.03, *p* = 0.15), distribution of lung shadows (bilateral or unilateral lungs, 90.3% vs. 97.6%, t = 4.98, *p* = 0.08), and radiological patterns of CIPs (t = 6.34, *p* = 0.09). There were more pleural abnormalities (including pleural effusion and thickening) in patients with high-grade CIPs (t = 10.39, *p* = 0.006).

There were more patients who were complicated with pulmonary infectious diseases in the high-grade CIPs group during follow-up (32.6% vs. 6.5%, t = 14.6, *p* = 0.02). However, there were no significant differences in all-cause mortality (t = 3.78, *p* = 0.05) between low-grade and high-grade CIPs groups. We also explored the survival of patients with low-grade vs. high-grade CIPs. If their survival was defined as the duration from the diagnosis of lung cancer, patients with low-grade CIPs showed a better prognosis than those with high-grade CIPs (χ^2^ = 5.07, *p* = 0.02). However, if their survival was defined as the duration from the diagnosis of CIPs, there was no statistical difference between them (χ^2^ = 3.19, *p* = 0.07).

## 4. Discussion

This retrospective study found that most patients with CIPs were older males, and the median onset time of CIPs was 2.77 months after the first dose of ICIs. Elevated serum IL-6, TNF-α, CRP, ESR, D-dimer, and LDH were common at the diagnosis of CIPs. Their chest CT manifestations coincided with the NSIP or OP pattern. In the high-grade CIPs group, patients were older and showed more fever, a higher ECOG score, a higher D-dimer, a lower serum albumin, more pulmonary infectious complications after CIPs, and a worse prognosis.

Demographic characteristics might be associated with the onset of irAE. Smoking, emphysema, and chronic obstructive pulmonary disease were the reported risk factors for CIPs. So, men were reported to be more likely to be complicated with CIPs in some studies [16,17,18]. However, as females were more susceptible to autoimmune disorders, female predominance was also reported in other studies [19]. In Jing’s meta-analysis, they reported that there were no significant differences in age distributions in patients with NSCLC when they suffered from irAEs based on clinical data, real-world pharmacovigilance data, and their hospital’s data [20]. On the other hand, older age was reported as a risk factor in several studies [20,21], which might be associated with upregulation of gene expression involved in the JNK cascade and collagen-containing extracellular matrix. In our study, although our enrolled CIPs were mainly older men, there were no significant differences in age distributions between the high-grade and low-grade CIPs. But lung cancer patients with high-grade CIPs were both older and had higher ECOG scores than those with low-grade CIPs. In clinical practice, we should keep a close eye on older and weaker lung cancer patients when they are prescribed ICIs.

Several cytokines or biomarkers were reported to be associated with the onset and/or severity of CIPs, including IL-6, IL-10, LDH, albumin, ESR, CRP, TNF-α, etc. [2,22,23]. However, their research results were inconsistent with each other. In Kowalski’s study, they analyzed bronchoalveolar lavage fluid (BALF) and serum concentrations of IL-6 for the same lung cancer patients before the occurrence of CIPs and after the onset of CIPs. They found that although BALF IL-6 concentration was elevated significantly as CIPs onset, there was no difference in serum IL-6 [23]. In Lin’s study, they analyzed a series of serum cytokines in different lung cancer patient groups with or without CIPs. It was shown that serum concentrations of IL-6, IL-10, and LDH were higher in the CIPs group when compared with the non-CIPs group. Higher serum IL-6 and lower albumin were associated with high-grade CIPs and a poor prognosis [23]. We only tested serum cytokines and biomarkers for our CIP patients when they were suffering from CIPs. Serum cytokine levels after treatment with anti-cancer ICIs before the development of CIPs were not tested. Regular detection of serum cytokines and biomarkers was suggested for patients with lung cancer during ICIs treatment. Serum IL-6 and TNF-α were elevated when they suffered from CIPs; however, serum IL-10 and IL-8 were not elevated significantly. As JAK inhibitors (JAKi) could effectively inhibit the release of IL-6 and TNF-α, JAKi and anti-TNF medications might be steroid-sparing medications for severe and/or refractory CIPs, besides tocilizumab [24,25].

As reported in previous studies, NSIP and/or OP radiological patterns were common chest CT patterns in CIP patients [6,26,27,28]. It is worth noting that these radiological features were similar to chest CT manifestations of SARS-CoV-2 infection-associated interstitial lung disease and/or COVID-19 pneumonia [29,30,31,32]. Furthermore, IL-6, IL-10, LDH, ESR, C-reactive protein, TNF-α, etc. were also commonly elevated in patients with COVID-19 pneumonia [33]. So, sometimes it was difficult to differentiate CIPs from COVID-19 pneumonia based on clinical and chest CT manifestations, especially when a throat swab COVID-19 antigen or nucleic acid test was negative. When COVID-19 pneumonia was highly suspected in lung cancer patients during ICIs therapy, viral RNA detection in BALF was suggested because of its higher sensitivity [34]. The SARS-CoV-2 infection was strictly controlled before 7 December 2022. All patients were arranged for SARS-CoV-2 RNA detection before every admission, and all of our enrolled CIP patients were SARS-CoV-2 RNA negative. However, during the SARS-CoV-2 epidemic era, COVID-19 pneumonia should be an important differential diagnosis of CIP.

Corticosteroids were the recommended medications for grade 2 to 4 CIPs, and add-ons infliximab, mycophenolate mofetil, tacrolimus, cyclophosphamide, and other immunosuppressants were suggested for steroid-refractory CIPs and/or high-grade CIPs [6,25,35,36]. Both ICIs and malignancies would decrease patients’ capacity for infection surveillance. On the other hand, corticosteroids and/or non-steroidal immunosuppressants that were administered for irAE would also be risk factors for severe infectious disease, especially opportunistic infectious disease [10,37]. In our cohort, complications with pulmonary infectious diseases were higher for high-grade CIPs. And those with poor prognosis after CIPs were more likely to be reported with pulmonary infectious disease. In our study, more patients with a worse prognosis were treated with IVIg, tocilizumab, antibiotics, and anti-fungal medications. On the other hand, we also showed that patients with a worse prognosis were prescribed a longer duration of large-dose corticosteroids. Therefore, optimized therapy with a shorter duration and/or a smaller dosage of corticosteroids might be explored for patients with high-grade CIPs, including JAK inhibitors, abatacept, and/or tocilizumab. JAK inhibitors or JAK inhibitor combinations with abatacept were reported to successfully treat life-threatening or refractory ICI-related myocarditis [38,39,40]. Prospective large-scale clinical trials about the efficacy and safety of JAK inhibitors for high-grade CIPs are expected in the future.

There are several limitations to this study. First, it was a retrospective study from a tertiary hospital, and all of the enrolled participants were hospitalized patients. All patients were diagnosed with lung cancer pathologically. These factors might lead to selection bias. Secondly, not all enrolled patients were arranged for serum cytokine analysis, and cytokines were not tested regularly after ICI treatment. So, we could not compare serum cytokines for lung cancer patients who were treated with anti-cancer ICIs vs. without CIPs. Thirdly, bronchoalveolar lavage fluid analysis and/or lung biopsy were not routinely performed for patients with lung cancer when they were suffering from CIPs. Features of BALF cell differentiation could not be analyzed in our study. Fourth, since our study was a retrospective study, the detailed ICIs and other anti-cancer therapy regimens varied from patient to patient. A multicenter, well-designed prospective study is expected in the future.

## 5. Conclusions

Compared with patients with low-grade CIPs, patients with high-grade CIPs were older, with higher ECOG scores, higher D-dimer, and more pulmonary infectious diseases after CIPs. Complications with pulmonary infectious diseases, management with IVIg, tocilizumab, antibiotics, and anti-fungal medications, and longer duration of large-dose corticosteroids were more common for CIP patients with a worse prognosis. As there were several limitations to our retrospective study, a well-designed multicenter prospective study is expected.

## Figures and Tables

**Table 1:** Management of CIPs with different outcomes.

Management	Group 1 * (*n* = 63)	Group 2 ** (*n* = 11)	χ^2^ or t Value	*p* Value
Duration of methylprednisolone > 80 mg/d (days)	3.14	8.82	3.39	0.001
Initial dosage of prednisone > 1 mg/kg/d (*n*/%)	35/55.6%	9/81.8%	2.68	0.18
Duration of prednisolone > 1 mg/kg/d (days)	9.7	18.5	3.26	0.002
Tocilizumab (*n*/%)	5/7.9%	4/36.4%	7.08	0.02
IVIg (*n*/%)	5/7.9%	10/90.9%	9.57	0.004
Antibiotics administration (*n*/%)	41/65.1%	11/100%	5.47	0.03
Anti-fungal administration (*n*/%)	5/7.9%	5/45.5%	11.3	0.005

*: Patients with CIPs recovered, improved, and/or remained stable after treatment. **: Patients with CIPs progressed and/or were dead. CIP: checkpoint inhibitor pneumonits; IVIg: intravenous immunoglobulin.

**Table 2:** The baseline characteristics between low-grade and high-grade CIPs.

Characteristics	Low-Grade CIP (*n* = 31)	High-Grade CIP (*n* = 43)	χ^2^ or t Value	*p* Value
Age (years) *	61.5 ± 10.1	65.8 ± 6.5	2.21	0.03
Sex (male%)	77.4%	79.1%	0.03	0.87
Smoking history (*n*/%) **	18/58.1%	30/69.8%	0.75	0.39
ECOG before ICIs therapy (*n*/%)				
0	18/58.1%	12/27.9%		
1	13/41.9%	28/65.1%	7.95	0.047
2	0	2/4.7%		
3	0	1/2.3%		
Underlying diseases (*n*%)				
none	17/54.8%	26/60.5%		
autoimmune diseases	0	1/2.3%	1.22	0.75
other malignancies	7/22.6%	7/16.3%		
diabetes	7/22.6%	9/20.9%		
Pathological patterns of lung cancer (*n*/%)				
adenocarcinoma	13/41.9%	19/44.2%		
squamous cell cancer	10/32.2%	21/48.8%	6.25	0.18
small cell lung cancer	4/12.9%	2/4.7%		
others	4/12.9%	1/2.3%		
Pre-existing ILDs (*n*/%)	4/12.9%	11/25.6%	1.62	0.20
Surgical resection (*n*/%)				
none	28/90.3%	36/83.7%		
lobe resection	3/9.7%	4/9.3%	2.26	0.32
wedge resection	0	3/7%		
Non-surgical treatment (*n*/%)				
radiotherapy (*n*/%)	11/35.5%	12/27.9%	0.48	0.49
molecular targeting therapy	7/22.6%	4/9.3%	0.02	0.11
chemotherapy	27/87.1%	37/86.0%	0.02	0.90
ICIs therapy (*n*/%)				
first line	14/45.2%	22/51.2%		
second line	10/32.2%	9/20.9%		
more than the second line	4/12.9%	3/7%	7.42	0.19
new adjuvant therapy	0	6/14%		
maintenance therapy	2/6.5%	3/7%		
post-surgery adjuvant therapy	1/3.2%	0		
With other irAEs (*n*/%)	7/22.6%	12/27.9%	0.27	0.61

*: as they suffered from CIP. **: both current and former smokers CIP: checkpoint inhibitor pneumonitis; ICI: immune checkpoint inhibitor; ILD: interstitial lung disease; irAE: immune-related adverse effect.

**Table 3:** The clinical characteristics of low-grade and high-grade CIPs.

Characteristics	Low-Grade CIP (*n* = 31)	High-Grade CIP (*n* = 43)	χ^2^ or t Value	*p* Value
ECOG as suffering from CIPs (*n*/%)				
0	2/6.5%	1/2.3%		
1	20/64.5%	9/20.9%		
2	5/16.1%	11/25.6%	17.9	0.001
3	4/12.9%	20/46.5%		
4	0	2/4.7%		
Fever	9/29%	28/65.1%	9.38	0.002
Laboratory analysis *				
albumin (g/L)	37.9 ± 4.6	35.2 ± 4.2	2.59	0.01
D-dimer (mg/L)	1.7 ± 2.2	5.1 ± 7.1	2.36	0.02
CRP (mg/L)	45.8 ± 48.1	68.5 ± 56.8	1.53	0.13
ESR (mm/h)	56.6 ± 32.3	63 ± 33.1	0.63	0.53
LDH (U/L)	286.1 ± 103.7	350.1 ± 157	1.74	0.09
Radiological patterns of CIP (*n*/%)				
NSIP pattern	12/38.7%	21/48.8%		
OP pattern	15/48.4%	15/34.9%	6.34	0.09
NSIP overlap OP pattern	0	5/11.6%		
Others	4/12.9%	2/4.7%		
Chest CT features (*n*/%)				
CIP in both lungs	28/90.3%	42/97.6%	4.98	0.08
Emphysema	10/32.2%	21/48.8%	2.03	0.15
lymphadenopathy **	6/19.4%	23/53.5%	8.81	0.003
pleural abnormalities				
pleural thickening	16/51.6%	22/51.2%		
pleural effusion	5/16.1%	18/41.9%	10.39	0.006
normal	10/32.2%	3/7%		
Complicated with infectious disease during CIP management (*n*/%)	2/6.5%	14/32.6%	14.62	0.02
Initial dosage of corticosteroids (>1 mg/kg/d ***, *n*/%)	20/64.5%	24/55.8%	0.57	0.45
Tocilizumab (*n*/%)	11/35.5%	13/30.2%	0.23	0.63
IVIg (*n*/%)	7/22.6%	8/18.6%	0.18	0.68
Outcomes (*n*/%)				
cured	0	3/7%		
improved	24/77.4%	30/72.1%		
stable	3/9.7%	3/7%	3.21	0.52
progressed	0	1/2.3%		
dead	4/12.9%	6/14%		
All-cause mortality (*n*/%)	4/12.9%	14/32.6%	3.78	0.05
ICIs rechallenge	9/29%	6/14%	2.53	0.11

*: the first laboratory test as suffering from CIPs. **: the short diameter of the chest lymph node. ***: the dosage of prednisone. CIP: checkpoint inhibitor pneumonitis, CRP: C-reactive protein, ESR: erythrocyte sedimentation rate, LDH: lactate dehydrogenase, NSIP: nonspecific interstitial pneumonia, OP: organizing pneumonia, IVIg: intravenous immunoglobulin, ICI: immune checkpoint inhibitor.

## Data Availability

The datasets generated during and/or analyzed during the current study are available from the corresponding author on reasonable request.
